# A multi‐institutional evaluation of small field output factor determination following the recommendations of IAEA/AAPM TRS‐483

**DOI:** 10.1002/mp.15797

**Published:** 2022-07-08

**Authors:** Wolfgang Lechner, Rodolfo Alfonso, Mehenna Arib, M. Saiful Huq, Anas Ismail, Rajesh Kinhikar, José M. Lárraga‐Gutiérrez, Karthick Raj Mani, Nkosingiphile Maphumulo, Otto A Sauer, Shaima Shoeir, Sivalee Suriyapee, Karen Christaki

**Affiliations:** ^1^ Department of Radiation Oncology, Division of Medical Physics Medical University Vienna Vienna Austria; ^2^ Department of Nuclear Engineering, Higher Institute of Technology and Applied Sciences University of Havana Havana Cuba; ^3^ King Faisal Specialist Hospital and Research Centre Riyadh Saudi Arabia; ^4^ Department of Radiation Oncology University of Pittsburgh School of Medicine and UPMC Hillman Cancer Center Pittsburgh Pennsylvania USA; ^5^ Protection and Safety Department Atomic Energy Commission of Syria Damascus Syria; ^6^ Department of Medical Physics Tata Memorial Centre, Mumbai, India 400012 & Homi Bhabha National Institute Mumbai India; ^7^ Laboratorio de Física‐Médica Instituto Nacional de Neurología y Neurocirugía Tlalpan, CDMX México; ^8^ Department of Radiation Oncology United Hospital Ltd. Dhaka Bangladesh; ^9^ Radiation Dosimetry Section National Metrology Institute of South Africa Pretoria South Africa; ^10^ Department of Radiation Oncology University of Würzburg Würzburg Germany; ^11^ Children's Cancer Hospital Egypt Cairo Egypt; ^12^ Division of Radiation Oncology, Department of Radiology Chulalongkorn University Bangkok Thailand; ^13^ International Atomic Energy Agency Vienna Austria

**Keywords:** small field dosimetry, small field output factors, TRS‐483

## Abstract

**Purpose:**

The aim of this work was to test the implementation of small field dosimetry following TRS‐483 and to develop quality assurance procedures for the experimental determination of small field output factors (SFOFs).

**Materials and methods:**

Twelve different centers provided SFOFs determined with various detectors. Various linac models using the beam qualities 6 MV and 10 MV with flattening filter and without flattening filter were utilized to generate square fields down to a nominal field size of 0.5 cm × 0.5 cm. The detectors were positioned at 10 cm depth in water. Depending on the local situation, the source‐to‐surface distance was either set to 90 cm or 100 cm. The SFOFs were normalized to the output of the 10 cm × 10 cm field. The spread of SFOFs measured with different detectors was investigated for each individual linac beam quality and field size. Additionally, linac‐type specific SFOF curves were determined for each beam quality and the SFOFs determined using individual detectors were compared to these curves. Example uncertainty budgets were established for a solid state detector and a micro ionization chamber.

**Results:**

The spread of SFOFs for each linac and field was below 5% for all field sizes. With the exception of one linac‐type, the SFOFs of all investigated detectors agreed within 10% with the respective linac‐type SFOF curve, indicating a potential inter‐detector and inter‐linac variability.

**Conclusion:**

Quality assurance on the SFOF measurements can be done by investigation of the spread of SFOFs measured with multiple detectors and by comparison to linac‐type specific SFOFs. A follow‐up of a measurement session should be conducted if the spread of SFOFs is larger than 5%, 3%, and 2% for field sizes of 0.5 cm × 0.5 cm, 1 cm × 1 cm, and field sizes larger than 2 cm × 2 cm, respectively. Additionally, deviations of measured SFOFs to the linac‐type‐curves of more than 7%, 3%, and 2% for field sizes 0.5 cm × 0.5 cm, 1 cm × 1 cm, and field sizes larger than 1 cm × 1 cm, respectively, should be followed up.

## 1 INTRODUCTION

Dosimetry in small and nonstandard photon fields has been a challenging topic in radiation oncology for decades. Small fields are frequently utilized in stereotactic (body) radiotherapy or intensity modulated radiotherapy.^1^ When treating small lesions, the utilization of these small fields is inevitable. As small field output factors (SFOFs) have a direct influence on the dose distribution and the calculation of monitor units, incorrectly measured or incorrectly calculated SFOFs directly translate into an incorrect prediction of the dose distribution of treatment plans. Modern intensity modulated treatment techniques for larger treatment volumes are a composition of broad and small fields. In this scenario, the effects of incorrectly measured or implemented SFOFs may remain undetected on a patient‐specific level. For treating smaller lesions, the impact of incorrectly measured SFOFs becomes more pronounced.^2^ These effects can also influence the results of dosimetry audits and need to be considered separately.^3^, ^4^, ^5^, ^6^

The determination of SFOFs is challenging as the ratio of detector readings is in general not equal to the ratio of dose.^1^, ^7^, ^8^ Additionally, the use of appropriate detectors and accurate positioning of these detectors in small fields are essential to minimize the experimental uncertainty.^9^

In a joint effort, the IAEA in collaboration with the AAPM published a code of practice on dosimetry of small static fields used in external beam radiotherapy. This code of practice (TRS‐483) provides a guideline on the determination of absorbed dose in small photon fields as well as detector and field size‐specific output correction factors for relative dosimetry.^7^, ^8^ Since then, a few studies on the application of TRS‐483 for determination of SFOFs for standard LINACs^10^, ^11^, ^12^, ^13^, ^14^ as well as correction factors for detectors or detector orientations that are not available in TRS‐483 have been published.^15^, ^16^, ^17^

In 2015, the International Atomic Energy Agency initiated a coordinated research project (Testing of Code of Practice on Small Field Dosimetry—E24021) to test the consistency of the procedures and correction factors published in TRS‐483 to find pitfalls, if any, and to guide Member States with its implementation. This work summarizes the findings of this working group on the determination of SFOFs and the associated uncertainties of small photon fields produced by standard LINACs. Detectors requiring no corrections of the raw output signal (except for volume averaging) in small fields, for example, plastic scintillators, are not widely available in clinical settings. TRS‐483 provides correction factors for commonly available detectors but benchmark data of different linac types or recommendations on the quality assurance of SFOF measurements are scarce. Therefore, experimental data on SFOFs were collected in a multi‐institutional setting for a representative set of different treatment units and detectors. Based on that, two methods for quality assurance of the experimental determination of SFOFs were developed.

## 2 MATERIALS AND METHODS

### 2.1 Linear accelerators and detectors

The equipment used by the participating centers is summarized in Table 1. The detector types used in this work are summarized in Table 2.

**TABLE 1:** Summary of treatment machines and beam energies per center

	Beam Energy			
Centre	6 MV WFF	6 MV FFF	10 MV WFF	10 MV FFF
1	Elekta Versa HD	Elekta Versa HD		Elekta Versa HD
2			Varian TrueBEAM	Varian TrueBEAM
3	Elekta Precise			
4	Elekta Versa HD	Elekta Versa HD	Elekta Versa HD	Elekta Versa HD
5	Siemens Primus		Elekta Versa HD	Elekta Versa HD
6	Varian TrueBEAM		Varian TrueBEAM	
7	Varian TrueBEAM Stx	Varian TrueBEAM Stx		
8	Siemens Primus			
9	Varian TrueBEAM			
10	Varian 21EX			
11	Varian TrueBEAM	Varian TrueBEAM	Varian TrueBEAM	Varian TrueBEAM
12	Varian TrueBEAM Stx	Varian TrueBEAM Stx	Varian TrueBEAM Stx	Varian TrueBEAM Stx

**TABLE 2:** Summary of detector types used per beam energy. Note that each center had its own set of detectors

Beam energy			
6 MV WFF	6 MV FFF	10 MV WFF	10 MV FFF
IBA EFD3G unshielded diode	IBA EFD3G unshielded diode	IBA EFD3G unshielded diode	IBA EFD3G unshielded diode
IBA PFD3G shielded diode	IBA PFD3G shielded diode	IBA PFD3G shielded diode	IBA PFD3G shielded diode
IBA SFD unshielded diode	IBA SFD unshielded diode	IBA/Wellhoefer CC01	IBA/Wellhoefer CC01
IBA/Wellhoefer CC01	IBA/Wellhoefer CC01	IBA/Wellhoefer CC13	IBA/Wellhoefer CC13
IBA/Wellhoefer CC13	IBA/Wellhoefer CC13	PTW 31014 PinPoint	PTW 60008 shielded diode
PTW 31006	PTW 31016 PinPoint 3D	PTW 60008 shielded diode	PTW 60012 unshielded diode
PTW 31010 Semiflex	PTW 60017 unshielded diode	PTW 60012 unshielded diode	PTW 60017 unshielded diode
PTW 31016 PinPoint 3D	PTW 60019 CVD diamond	PTW 60017 unshielded diode	PTW 60019 CVD diamond
PTW 31018 liquid ion chamber	Sun Nuclear EDGE detector	PTW 60019 CVD diamond	Sun Nuclear EDGE detector
PTW 60008 shielded diode		Sun Nuclear EDGE detector	
PTW 60012 unshielded diode			
PTW 60017 unshielded diode			
PTW 60019 CVD diamond			
Sun Nuclear EDGE detector			

### 2.2 Experimental setup

Experimental data acquired by centers in Austria, Bangladesh, Cuba, Egypt, Germany, India, Mexico, Saudi Arabia, South Africa, Syria, Thailand, and USA were collected for this work. In this work, the nominal beam energies 6 MV and 10 MV with flattening filter (WFF) and without flattening filter (FFF) were used to determine SFOFs for the following nominal field sizes: 10 cm × 10 cm, 6 cm × 6 cm, 4 cm × 4 cm, 3 cm × 3 cm, 2 cm × 2 cm, 1 cm × 1 cm, and the smallest field size available (0.5 cm × 0.5 cm or 0.6 cm × 0.6 cm) shaped with the multi‐leaf collimator (MLC). The centers used their detectors suitable for the given field sizes. All centers provided data for the 6 MV WFF beam energy, which was considered the pilot dataset for testing the implementation of the methodology. For the other beam energies, only a subset of the participating centers provided data. The detectors were positioned at 10 cm depth in water. Depending on the local practice, the centers either used source‐to‐axis (SAD) or source‐to‐surface distance (SSD) setup of 100 cm. Standard measurement guidelines were given to all participants so that the measured data results from uniform measurement procedures across the centers. The detectors were initially positioned using the room lasers or the linac's crosshair. This initial position was optimized prior to the measurement of the SFOFs by acquiring the lateral beam profiles of that particular field and re‐positioning of the detector at the position of the maximum signal, if necessary. A small step size (0.1 mm or 0.2 mm) and a slow scanning speed (< = 0.2 mm/s) were used for the acquisition of the beam profiles. Based on these profile measurements, the equivalent square small field size $S_{\mathrm{clin}}=\sqrt{\mathrm{AB}}$ was determined. *A* and *B* are the in‐plane and cross‐plane dosimetric field widths defined as the full‐width‐at‐half‐maximum (FWHM) of the beam profile at the same measurement depth as for the SFOFs of 10 cm. The same definition was used for WFF and FFF beams as the beam profiles of small fields are similar.^18^ At least three consecutive readings were acquired per field. The ratios of readings were corrected using the detector and field size specific $k_{Q_{clin},Q}^{f_{clin},f_{ref}}$ factors listed in TRS‐483^7^ and determined using $S_{\mathrm{clin}}$ measured with each detector. All SFOFs were normalized to the reference 10 cm × 10 cm field ($f_{\mathrm{ref}})$.

### 2.3 Data analysis

To investigate the SFOFs of the different linac types used in this work, all SFOFs were plotted as a function of $S_{\mathrm{clin}}$ determined with the respective detector. The function proposed by Sauer and Wilbert^19^ was fitted to these SFOFs. For the fitting procedure, the data were grouped according to linac type and only nominal field sizes with SFOFs determined with at least two different detectors were considered. The resulting functions are henceforth referred to as linac‐type‐curves and the following function was used for fitting:

(1) $$\Omega_{Q_{\mathrm{clin}},Q}^{f_{\mathrm{clin}},f_{\mathrm{ref}}}\left(S_{\mathrm{clin}}\right)=P_{\infty }\frac{{S_{\mathrm{clin}}}^{n}}{l^{n}+{S_{\mathrm{clin}}}^{n}}+S_{\infty }\left(1-e^{-b\cdot S_{\mathrm{clin}}}\right)$$

$P_{\infty }$and $S_{\infty }$ can be interpreted as the primary and scattered dose contribution of an infinitely large field. $S_{\mathrm{clin}}$is the equivalent square small field size. The parameter *b* describes the increase of the scatter contribution with increasing field size. The parameters *n* and *l* are model parameters without deeper physical meaning. Tolerance levels of the linac‐type‐curves were calculated as two times the standard deviation of residuals of the fitting procedure including all linac types and energies.

Additionally, all SFOFs $\Omega_{Q_{\mathrm{clin}},Q}^{f_{\mathrm{clin}},f_{\mathrm{ref}}}$ were grouped for each center, linac, beam energy, and nominal field size. This resulted in sets of SFOFs containing two or three values for each center, linac, beam energy, and nominal field size. The spread of a set of SFOFs was defined as

(2) $$\begin{array}{ccc} & & \mathrm{sprd}\left(\left\{\Omega_{Q_{\mathrm{clin}},Q}^{f_{\mathrm{clin}},f_{\mathrm{ref}}}\right\}\right)\\ & & =\frac{\max\left(\left\{\Omega_{Q_{\mathrm{clin}},Q}^{f_{\mathrm{clin}},f_{\mathrm{ref}}}\right\}\right)-\min\left(\left\{\Omega_{Q_{\mathrm{clin}},Q}^{f_{\mathrm{clin}},f_{\mathrm{ref}}}\right\}\right)}{\mathrm{mean}\left(\left\{\Omega_{Q_{\mathrm{clin}},Q}^{f_{\mathrm{clin}},f_{\mathrm{ref}}}\right\}\right)} \end{array}$$

where $\left\{\Omega_{Q_{\mathrm{clin}},Q}^{f_{\mathrm{clin}},f_{\mathrm{ref}}}\right\}$ is a set of SFOFs determined for a particular nominal field size $f_{\mathrm{clin}}$, beam energy, linac, and center. This spread was then plotted as a function of the mean value of $S_{\mathrm{clin}}$ for this particular set of SFOFs. The resulting figures are henceforth referred to as spread plots. It needs to be highlighted that this analysis is not influenced by inter‐linac variation with respect to the SFOFs.

### 2.4 Assessment of uncertainties

Two representative uncertainty budgets were produced based on two different detector types used in this study. The following model equation was used to calculate the SFOFs:

(3) $$\Omega_{Q_{\mathrm{clin}},Q}^{f_{\mathrm{clin}},f_{\mathrm{ref}}}=\frac{M_{Q_{\mathrm{clin}}}^{f_{\mathrm{clin}}}}{M_{Q_{\mathrm{ref}}}^{f_{\mathrm{ref}}}}k_{Q_{\mathrm{clin}},Q}^{f_{\mathrm{clin}},f_{\mathrm{ref}}}k_{\mathrm{FS}}k_{\mathrm{pos}}k_{\mathrm{drift}}k_{\mathrm{other}}$$

The correction factors $k_{\mathrm{FS}}$, $k_{\mathrm{pos}}$, $k_{\mathrm{drift}}$ and $k_{\mathrm{other}}$ were assumed to be unity and were introduced for formal reasons to account for the uncertainties of the respective processes or quantities. The uncertainty of the determination of $S_{\mathrm{clin}}$ and a possible change of the field size between determination of $S_{\mathrm{clin}}$ and the actual output factor measurement is summarized as uncertainty of $k_{\mathrm{FS}}$. For that, an uncertainty of 0.1 mm for the determination and drift of the field size was used. The gradient of the fitting function describing the field output factor as a function of field size (see Equation (1) was used as sensitivity coefficient. For a field size in the order of 0.5 cm x 0.5 cm, this sensitivity coefficient is approximately 23%/mm. The uncertainty of $k_{\mathrm{pos}}$ represents the uncertainty of positioning the detector with respect to the center of the beam and was calculated based on a formalism proposed by Lechner et al.^9^ The uncertainty of $k_{Q_{\mathrm{clin}},Q}^{f_{\mathrm{clin}},f_{\mathrm{ref}}}$was selected from Table 37 in TRS‐483^7^. The uncertainty of $k_{\mathrm{drift}}$ summarizes uncertainties due to drifts of the detector signal and linac output. The uncertainty of $k_{\mathrm{other}}$ summarizes uncertainties of the measurement depth and SSD as well as drifts of temperature and pressure. Also, the uncertainty due to determination of the field size and positioning in the reference field are considered in this component. The uncertainty budgets for the determination of SFOFs using a PTW 600019 microDiamond and an IBA/Wellhöfer CC01 are provided in Tables 3 and 4, respectively.

**TABLE 3:** An example uncertainty budget for the determination of SFOFs using a PTW 60019 microDiamond

		Relative standard uncertainty		
Physical quantities or procedure	Type	0.5 cm x 0.5 cm	1 cm x 1 cm	≥2 cm x 2 cm
**Reference field**				
Dosimeter reading in ref. field	A	0.1%	0.1%	0.1%
**Clinical field**				
Dosimeter reading in clinical field	A	0.2%	0.2%	0.2%
Influence quantities in clinical field				
$k_{FS}$	B	2.3%	0.5%	0.1%
$k_{pos}$	B	0.4%	0.2%	0.1%
$k_{drift}$	B	0.3%	0.3%	0.3%
$k_{Q_{clin},Q}^{f_{clin},f_{ref}}$	B	0.8%	0.5%	0.4%
$k_{other}$	B	0.1%	0.1%	0.1%
**Combined standard uncertainty (coverage factor = 1)**	**combined**	**2.5%**	**0.9%**	**0.6%**

**TABLE 4:** An example uncertainty budget for the determination of SFOFs using an IBA/Wellhöfer CC01

		Relative standard uncertainty		
Physical quantities or procedure	Type	0.6 cm x 0.6 cm	1 cm x 1 cm	≥2 cm x 2 cm
**Reference field**				
Dosimeter reading in ref. field	A	0.1%	0.1%	0.1%
**Clinical field**				
Dosimeter reading in clinical field	A	0.2%	0.2%	0.2%
Influence quantities in clinical field				
$k_{FS}$	B	2.1%	0.5%	0.1%
$k_{pos}$	B	0.7%	0.4%	0.1%
$k_{drift}$	B	0.3%	0.3%	0.3%
$k_{Q_{clin},Q}^{f_{clin},f_{ref}}$	B	2.5%	1.1%	0.4%
$k_{other}$	B	0.1%	0.1%	0.1%
**Combined standard uncertainty (coverage factor = 1)**	**combined**	**3.4%**	**1.4%**	**0.6%**

## 3 RESULTS

The mean (range) of TPR_20,10_ of the beams used in this study were 0.672 (0.665–0.682), 0.649 (0.630–0.678), 0.736 (0.722–0.746), and 0.715 (0.706–0.723) for 6 MV WFF, 6 MV FFF, 10 MV WFF, and 10 MV FFF, respectively. The 6 MV FFF beam showed the largest variation of TPR_20,10_ values (7.4% for the FFF beams compared to 2.5% of the WFF beams) among the investigated beam energies.

**FIGURE 1:**
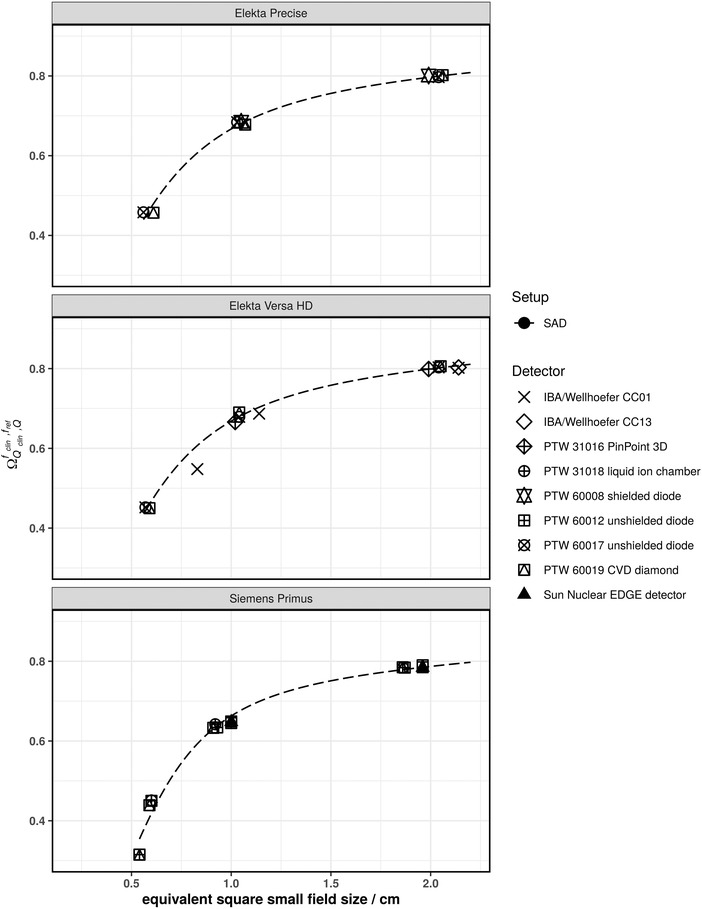
SFOFs for nominal field sizes ranging from 0.5 cm x 0.5 cm to 2 cm x 2 cm for a nominal beam energy of 6 MV WFF for three different types of linacs: Elekta Precise, Elekta Versa HD, and Siemens Primus. The linac‐type‐curves are depicted as dashed lines

**FIGURE 2:**
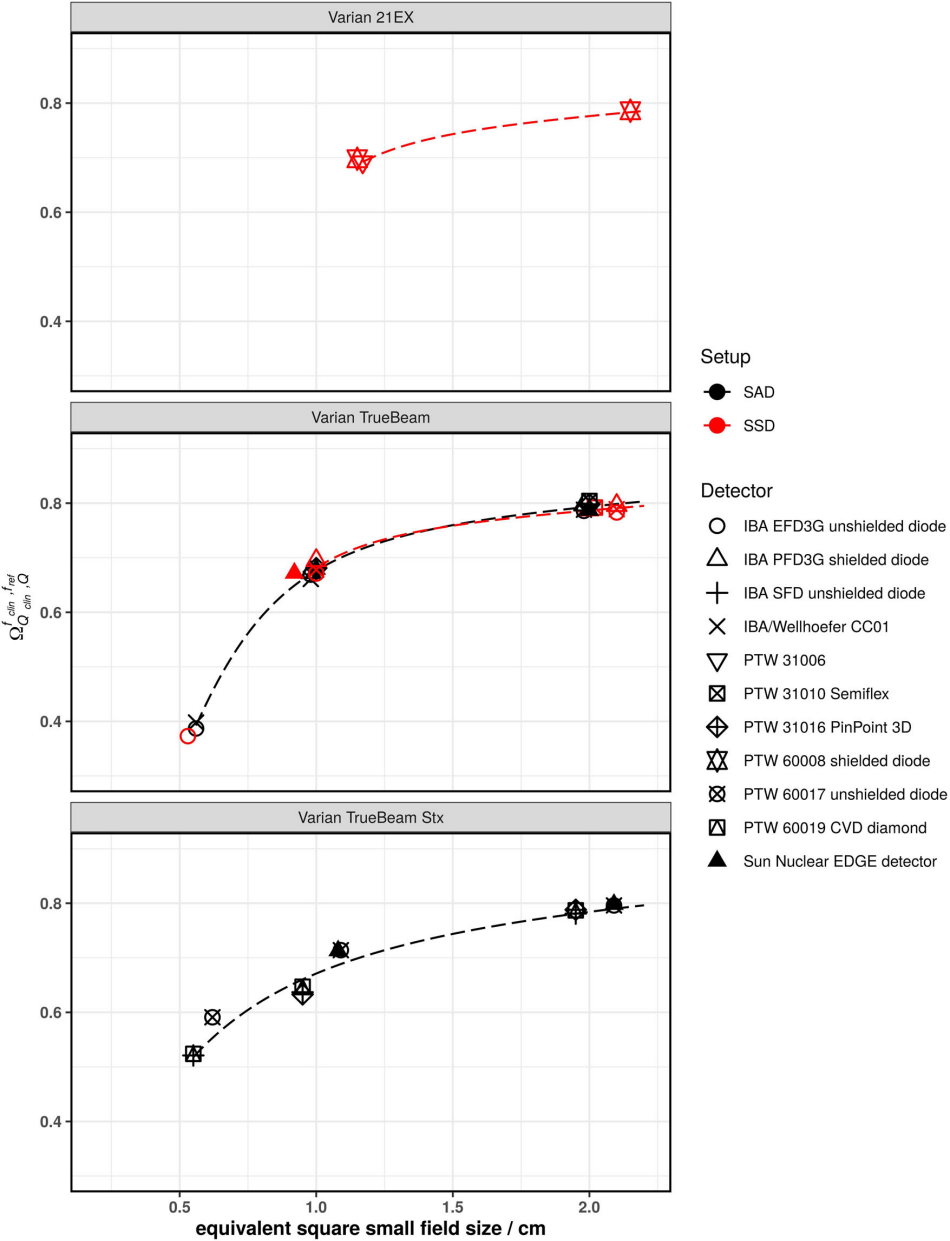
SFOFs for nominal field sizes ranging from 0.5 cm x 0.5 cm to 2 cm x 2 cm for a nominal beam energy of 6 MV WFF for three different types of linacs: Varian 21EX, Varian TrueBeam, and Varian TrueBeam Stx. The linac‐type‐curves are depicted as dashed lines

**FIGURE 3:**
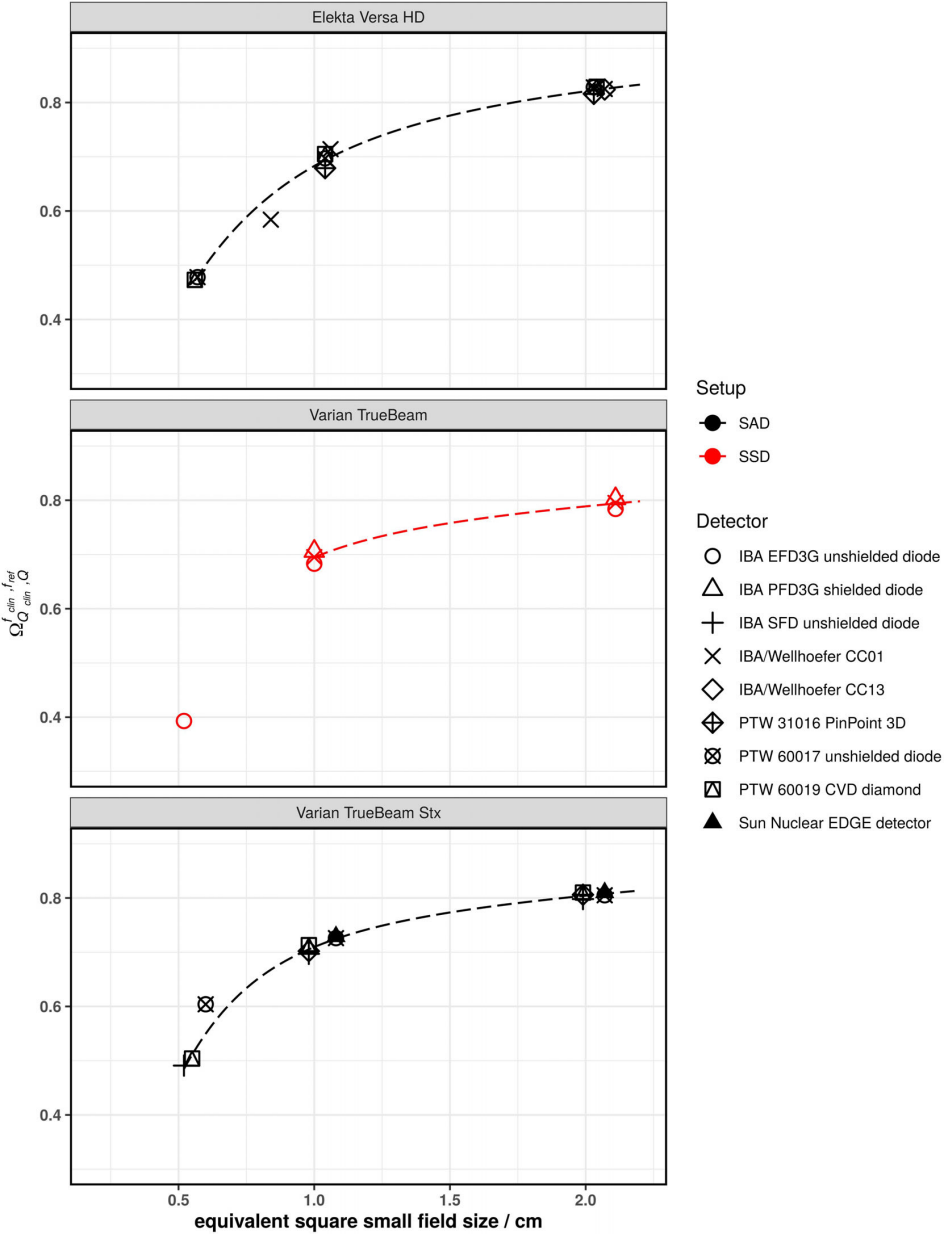
SFOFs for nominal field sizes ranging from 0.5 cm x 0.5 cm to 2 cm x 2 cm for a nominal beam energy of 6 MV FFF for three different types of linacs: Elekta Versa HD, Varian TrueBeam, and Varian TrueBeam Stx. The linac‐type‐curves are depicted as dashed lines

**FIGURE 4:**
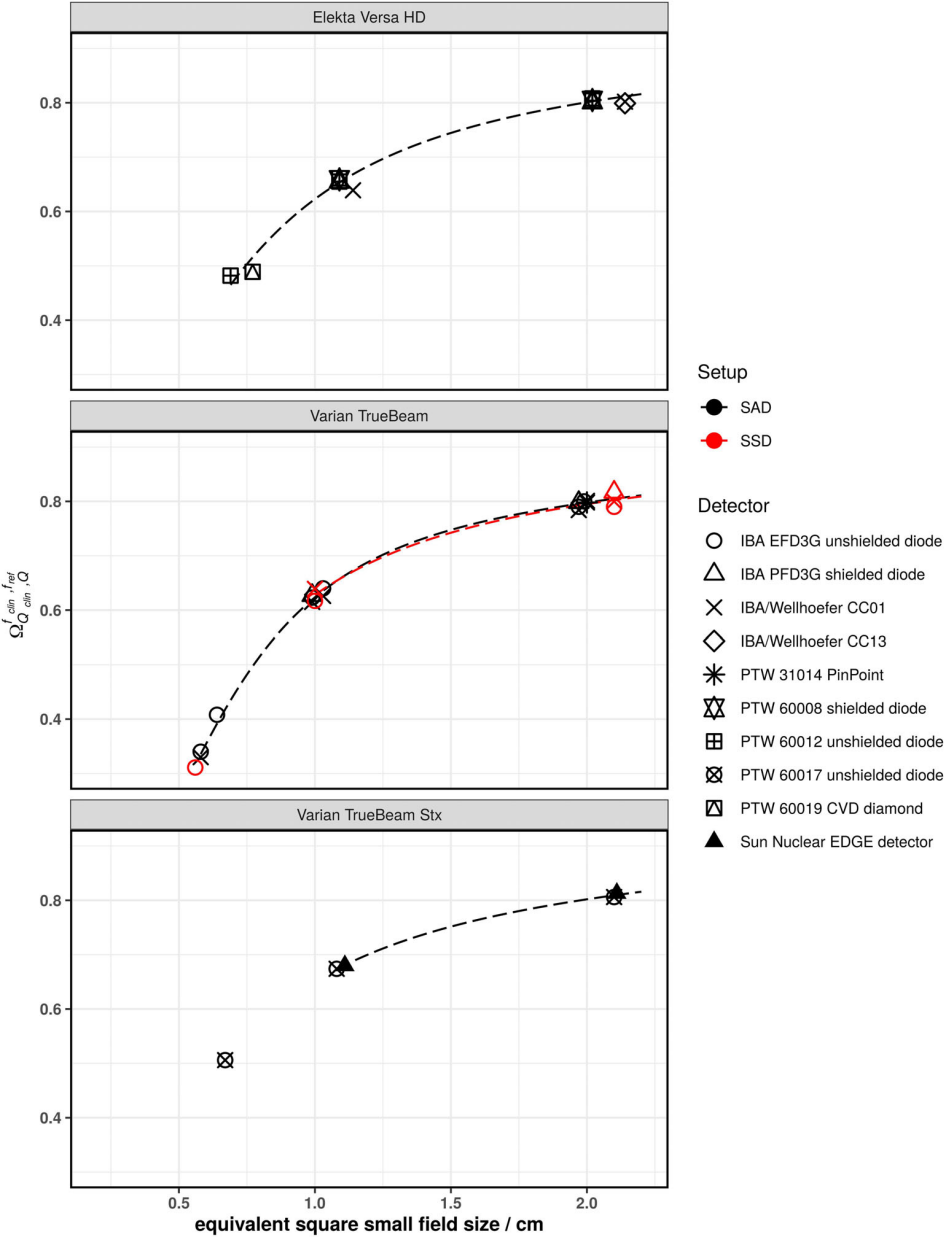
SFOFs for nominal field sizes ranging from 0.5 cm x 0.5 cm to 2 cm x 2 cm for a nominal beam energy of 10 MV WFF for three different types of linacs: Elekta Versa HD, Varian TrueBeam, and Varian TrueBEAM Stx. The linac‐type‐curves are depicted as dashed lines

**FIGURE 5:**
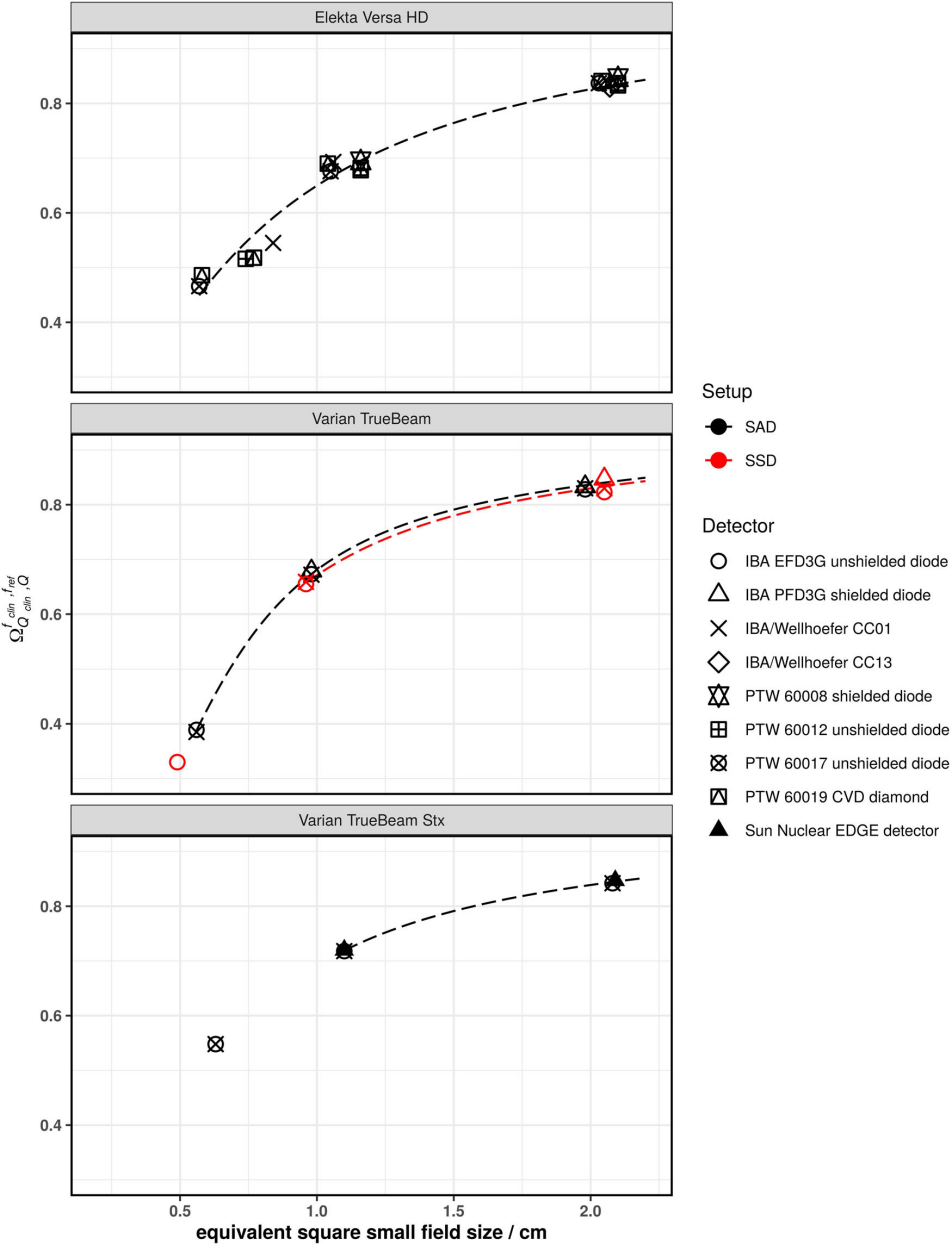
SFOFs for nominal field sizes ranging from 0.5 cm x 0.5 cm to 2 cm x 2 cm for a nominal beam energy of 10 MV FFF for three different types of linacs: Elekta Versa HD, Varian TrueBEAM, and Varian TrueBEAM Stx. The linac‐type‐curves are depicted as dashed lines

Figures 1, 2, 3, 4, 5 show the SFOFs determined with individual detectors for all energies separated by linac type as well as the resulting fits of Equation (1) to the data. The resulting fitting parameters are summarized in the supplementary material in Table S1.

The largest amount of data was available for 6 MV WFF beams. For field sizes of 1 cm × 1 cm and larger, all measured SFOFs agreed with the respective linac‐type‐curves to within 4%. For the 0.6 cm × 0.6 cm field, the largest deviations from a linac‐type‐curve were found for a Siemens Primus. For one center the measured field output factors were systematically lower by approximately 11% and for another center, the measured field output factors were systematically higher by approximately 7.7% compared to the linac‐type‐curve of a Siemens Primus. For the other linac types, two detectors deviating by more than 5% from the linac‐type‐curve were found. One was an IBA/Wellhöfer CC01 used with an Elekta Versa HD linac deviating by 9.7% and the other was a PTW 60017 used with a Varian TrueBeam Stx deviating by 6.9% from the respective linac‐type‐curves. Similar deviations for these two detectors were found for 6 MV FFF beams. In that case, the PTW 60017 showed a deviation of 9.9% to the TrueBeam STX linac‐type‐curve and the IBA/Wellhöfer CC01 showed a deviation of 7.1% to the Versa HD linac‐type‐curve. Both detectors were not considered for the fitting procedure of the 6 MV WFF and 6 MV FFF beams as these were only single measurements of the smallest fields of the respective linacs. The rest of the measured SFOFs agreed with the respective linac‐type‐curves to within 2.1% for 6 MV FFF.

With the exception of one outlier (IBA/Wellhöfer CC01 at Versa HD, 4.4 % deviation), which was not included in the fitting procedure as this field was measured with one detector only, the measured SFOFs agreed with the linac‐type‐curves to within 1.8% for 10 MV WFF beams and field sizes of 1 cm × 1 cm and larger. For the 0.5 cm × 0.5 cm field, all determined SFOFs agreed within 5.2% with the respective linac‐type‐curve.

The largest discrepancies between individually measured SFOFs and linac‐type‐curves were found for 10 MV FFF beams. The 0.5 cm × 0.5 cm fields for two individual Versa HD linacs were measured with different PTW 60019 detectors. One center reported the SFOF 7.5% below and another center reported an SFOF 6.1% above the respective linac‐type‐curve. Both of these measurement points were included in the fitting procedure since other measurements with other detectors supported these results. For the 1 cm × 1 cm field, the measurement of the SFOF with a PTW 60019 and an IBA/Wellhöfer CC01 deviated by 4.2% and 3.7%, respectively. The rest of the SFOFs agreed with those of the corresponding linac‐type‐curves to within 2.5%.

Most of the data was acquired using SAD setup. Two centers provided data acquired using the SSD setup and 6 MV WFF beams and one center for 6 MV FFF, 10 MV WFF, and 10 MV FFF beams. The linac‐type‐curves are provided for field sizes of 1 cm × 1 cm and larger since correction factors for the 0.5 cm × 0.5 cm field were only available for one detector. The individually measured SFOFs agreed with the linac‐type‐curves to within 2%. Naturally, these data points agreed very well with the fitted linac‐type‐curves due to the limited amount of data.

**FIGURE 6:**
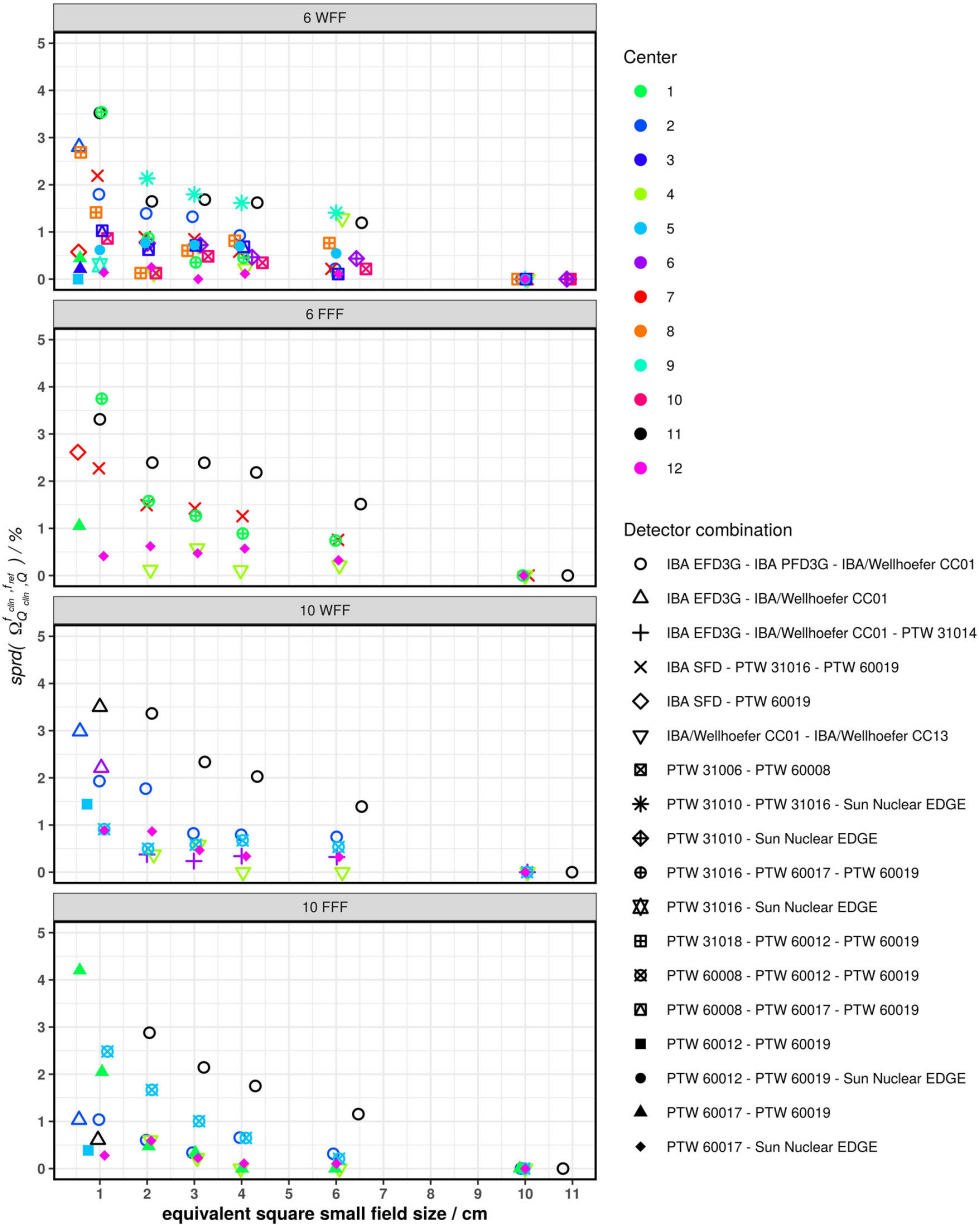
Spread plots of all investigated beam energies. Each data point represents the spread of the SFOFs of one linac for a given energy and nominal field size measured using two or three different detectors. Thereby this plot is not influenced by the inter‐linac deviation of the SFOFs. This relative spread of each detector combination is depicted for the following energies 6 MV WFF, 6 MV FFF, 10 MV WFF, and 10 MV FFF. The detector combination IBA/Wellhöfer CC01–IBA EFD–IBA PFD was of particular interest as the same combination was used by two different centers (Nr. 2 and 11). The spread measured by Centre 11 was systematically larger compared to the spread measured by Centre 2 for the 10 MV WFF and FFF beams

The spread plots of the SFOFs of all four investigated beam energies are shown in Figure 6. The spread of SFOFs increased with decreasing field sizes. For all beam energies, detector combinations, and field sizes, the spread was below 5%. A spread larger than 3% was observed for the detector combination IBA/Wellhöfer CC01–IBA EFD–IBA PFD (6 MV WFF, 6 MV FFF, 10 MV WFF), PTW 31016–PTW 60017–PTW 60019 (6MV WFF, 6 MV FFF), IBA/Wellhöfer CC01–IBA EFD (10MV WFF) and PTW 60017–PTW 60019 (10 MV FFF). For the detector combination IBA/Wellhöfer CC01–IBA EFD–IBA PFD and field sizes larger than 1 cm × 1 cm the spread measured by center 11 was systematically larger compared to other detector combinations for the beam energies 6 MV FFF, 10 MV WFF, and 10 MV FFF. This systematic offset was only observed by Centre 11 and not by Centre 2 using the same detector combination.

## 4 DISCUSSION

In this work, a comprehensive set of SFOFs determined using a large variety of different detector types for various linac types was collected and analyzed.

The spread of SFOFs determined for each linac, beam energy, and field size was below 5%. This is within the expected uncertainty with a coverage factor of two (*k* = 2) for a 0.5 cm × 0.5 cm field. For field sizes of 1 cm × 1 cm and larger, spreads larger than 3% were found for the detector combination shielded diode–unshielded diode–mini ionization chamber. For one center, an overestimation of the IBA PFD3G compared to the IBA EFD3G and IBA/Wellhöfer CC01 was observed for 6 MV WFF, 10 MV WFF, and 10 MV FFF beam qualities. This was systematic for the investigated energies and was confirmed by an additional measurement in a 10 MV FFF beam (see supplementary Figure S2). Moreover, the over‐response of the PDF3G was more than 5% compared to the other two detectors for the 1 cm × 1 cm field. Interestingly, another center using the same detector combination observed a lower spread. Since the over‐response of this individual PFD3G was systematic, reproducible, and can also not be explained by misalignment, the detector was removed from the analysis of the 1 cm × 1 cm field. A hardware fault or design changes could be a reason for this effect, but further investigation is necessary.

The detector combination PTW 31016–PTW 31017–PTW 31019 also showed a spread larger than 3% for the two FFF energies. In that case, the PTW 31016 underestimated the SFOF compared to the solid‐state detectors. Other centers using the PTW 31016 in combination with solid state detectors found a lower spread. Therefore, the observed spread was attributed to slight misalignment of the PTW 31016 chamber used for these measurements. This was confirmed by an additional measurement session using a 6 MV FFF beam and shown in supplementary Figure S3. In this context, it is worth highlighting that positioning an ionization chamber perpendicular to the beam's central axis is more challenging compared to the parallel orientation. Casar et al. recently published an extensive dataset of small field correction factors for ionization chambers in perpendicular and parallel orientation.^17^ The availability of correction factors for ionization chambers in parallel orientation make a change of the detector orientation between determination of $S_{\mathrm{clin}}$ and the actual SFOF measurement obsolete. However, confirmation of these correction factors is necessary.

These examples show that, investigating the spread of SFOFs measured with different detector types provides a quick and easy method for quality assurance of SFOF measurements. Provided that the measured equivalent square small field sizes are consistent for the investigated detectors (within 0.1 mm for field sizes smaller than 1 cm × 1 cm), a spread of more than 5%, 3%, and 2% for field sizes of 0.5 cm × 0.5 cm, 1 cm × 1 cm, and 2 cm × 2 cm and larger, respectively, should be followed up. An alternative to describing the variation of SFOFs measured with different detectors for a given linac, energy, and field is to express it as relative standard deviation, which is provided in the supplementary Figure S4.

Another way to analyze the data was to group them according to linac type and calculate a reference SFOF curve as a function of the equivalent square small field size. The fitting parameters provided in supplementary Table S1 allow the calculation of reference SFOFs for arbitrary equivalent square fields using Equation (1). The majority of SFOFs for field sizes of 1 cm × 1 cm and larger agreed to within 2% with those of the respective linac‐type‐curves. A few data points showed deviations of larger than 2% from the respective linac‐type‐curves but lower than 5% for the 1 cm × 1 cm field. Deviations of approximately 7% were found for the smallest field size of 0.5 cm × 0.5 cm (see Varian TrueBeam Stx in Figures 2 and 3 as well as Elekta Versa HD in Figure 5). These data points cannot be discarded as outliers as they could also be due to an inter‐linac variation. A deviation of SFOFs measured for various linacs of 5% to up to 17% were found by other groups.^11^, ^13^, ^20^ If the linac‐type‐curves derived in this work are used as benchmarks for SFOF measurements, an uncertainty with a coverage factor of two of at least 7%, 3%, and 2% for field sizes 0.5 cm × 0.5 cm, 1 cm × 1 cm and field sizes larger than 1 cm × 1 cm, respectively, needs to be considered. This tolerance was calculated based on the residuals of the fits calculated for the linac‐type‐curves. A plot of the residuals of the fitting procedure of all investigated linac types is provided as supplementary Figure S1. Only the 0.6 cm × 0.6 cm field of the Siemens Primus was excluded from this assessment as the residuals were substantially larger compared to the other linac types. Although the deviations of the measured data compared to the linac‐type‐curves were low for some linac types, one needs to bear in mind that the linac‐type‐curves are examples based on only two linacs and may be not be representative for the whole series of the same type. Therefore, the residuals of all linac‐type‐curves were used to assess the tolerance limits. Data points outside the proposed tolerance limits should be carefully followed up. Talamonti et al. suggested a deviation of 5% from their crowd knowledge‐based curve for checking small field measurements. Note that the crowd knowledge curve was derived for several linac types using the ratio of readings measured with the IBA Razor diode only, which is not listed in TRS‐483. Additionally, the measurements to establish this crowd knowledge‐based curve were conducted with two individual IBA Razor diodes. The larger tolerance level of 7% found in our work is likely due to the use of a larger variety of detectors.

Approximately 67% of the centers participating in the present study were also participating in clinical trials involving stereotactic radiotherapy and/or intensity modulated radiotherapy techniques where accurate dosimetry of small fields is essential. The proposed linac‐type‐curves along with the proposed tolerance limits can be used as initial quality assurance for the accreditation for clinical trials followed my more detailed experimental procedures.^4^, ^5^, ^6^

Unfortunately, the larger inter‐linac variation of the Siemens Primus could not be followed up as the machines were decommissioned. When operating this linac type with field sizes smaller than 1 cm x 1 cm, a larger inter‐linac variation might be expected compared to state‐of‐the‐art linacs and frequent checks of the SFOFs are necessary.

In this work, only detectors with correction factors published in TRS‐483 were considered, leading to the question of how to proceed with new detectors not covered by the current version of TRS‐483. Detectors suitable for small field dosimetry, which are not yet in TRS‐483, should only be used in combination with detectors requiring no corrections for small field effects other than a volume averaging correction and/or with detectors with correction factors found in TRS‐483. The consistency of the SFOFs measured with an unlisted detector with the SFOFs determined with detectors with the correction factor published in TRS‐483 needs to be investigated carefully. For example, the SFOF measured with an unlisted detector can be compared to a function fitted to the SFOF measured with detectors found in TRS‐483. This fitting function can be used to determine correction factors for the unlisted detector or detector orientation as done by Casar et al.^16^, ^17^ New publications on field output correction factors of unlisted detectors or detector orientations are necessary for further updates of TRS‐483 or other codes of practices.

A limitation of this work is that the determination of *S*_*clin*_ was not possible for all detectors. The main reason for that was the limited availability of the investigated treatment units and the time‐consuming process of determination of *S*_*clin*_ using ionization chambers. In that case, the centers reported *S*_*clin*_ measured by one high‐resolution solid‐state detector. The variation of *S*_*clin*_ for different linac types measured by different centers using different detector combinations is presented in supplementary Table S2. In that context, the importance of using *S*_*clin*_ in the dosimetry of small photon beams is frequently underestimated. It is worth mentioning here that *S*_*clin*_ is used to lookup the output correction factors and has therefore a direct influence on the SFOF. The FWHM should be measured with a high‐resolution detector at the measurement depth of the SFOF and *S*_*clin*_ must be calculated using the respective equations given in the TRS‐483. The whole process of determination of SFOFs is complicated and time‐consuming. Ideally, the work should be done by two persons independently or at least be reviewed by a second person. Prior to starting, the team should understand and discuss the measurement protocol based on TRS‐483. Only high‐quality data will help reducing the uncertainty of SFOF measurements.

## 5 CONCLUSION

Investigating the spread of SFOFs determined with different detectors can serve as a tool for quality assurance of SFOF measurements. A follow‐up of a measurement session should be conducted if the spread of SFOFs is larger than 5%, 3%, and 2% for field sizes of 0.5 cm × 0.5 cm, 1 cm × 1 cm, and field sizes larger than 2 cm × 2 cm, respectively. The presented linac‐type‐curves may be used for coarse consistency checks, but a possible inter linac variability needs to be considered. Deviations of measured SFOFs to the respective linac‐type‐curves above 7%, 3%, and 2% for field sizes 0.5 cm × 0.5 cm, 1 cm × 1 cm, and field sizes larger than 1 cm × 1 cm, respectively, should be followed up. For an effective use of these quality assurance methods, at least two different detector types need to be used for the experimental determination of SFOFs.

## CONFLICT OF INTERESTS

The authors have no conflicts to disclose.

## Supporting information

Supplementary material

Supplementary material

Supplementary material

Supplementary material

Supplementary material

Supplementary material

## Data Availability

The data can be made available upon request to the corresponding author.
