# Supplementary material for: A multi‐institutional evaluation of small field output factor determination following the recommendations of IAEA/AAPM TRS‐483
Source: Med Phys. 2022 Jul 8;49(8):5537–50. doi: 10.1002/mp.15797 (PMC9541513; doi:10.1002/mp.15797)
Supplement: Supplementary file 1 — Supplementary material [file MP-49-5537-s002.pdf]

Table S1 Summary of fit parameters per beam energy and linac type

			Parameters					Standard uncertainty of fitting parameters					Range for field sizs	
Energy	Setup	Linac Type	P	n	l / cm	S	b / cm^{-1}	u(P)	u(n)	u(l) / cm	u(S)	u(b) / cm^{-1}	Sclin min / cm	Sclin max / cm
**6 MV WFF**														
	SAD	Elekta Precise	0.760	2.409	0.512	0.435	0.081	0.028	0.210	0.010	0.117	0.045	0.56	10
	SAD	Elekta Versa HD	0.751	2.503	0.518	0.381	0.106	0.012	0.106	0.005	0.023	0.019	0.57	10
	SAD	SIEMENS Primus	0.716	3.268	0.566	0.444	0.103	0.023	0.234	0.008	0.069	0.038	0.54	10
	SAD	Varian TrueBEAM	0.727	3.297	0.555	0.453	0.092	0.008	0.101	0.004	0.033	0.015	0.56	10
	SAD	Varian TrueBEAM Stx	0.781	1.585	0.380	0.500	0.059	0.049	0.259	0.018	0.301	0.063	0.55	10
	SSD	Varian 21EX	0.688	5.000	0.711	0.423	0.122	0.015	3.669	0.238	0.011	0.017	1.2	11
	SSD	Varian TrueBEAM	0.710	4.399	0.607	0.425	0.105	0.019	3.275	0.205	0.026	0.023	1.0	11
**6 MV FFF**														
	SAD	Elekta Versa HD	0.811	2.041	0.496	0.312	0.095	0.019	0.105	0.007	0.038	0.032	0.56	10
	SAD	Varian TrueBEAM Stx	0.743	2.629	0.429	0.403	0.102	0.010	0.123	0.004	0.025	0.016	0.52	10
	SSD	Varian TrueBEAM	0.720	2.792	0.443	0.401	0.110	0.063	3.600	0.369	0.041	0.062	1.0	11
**10 MV WFF**														
	SAD	Elekta Versa HD	0.798	2.250	0.624	0.334	0.093	0.031	0.189	0.011	0.062	0.048	0.69	10
	SAD	Varian TrueBEAM	0.740	2.960	0.649	0.348	0.136	0.009	0.066	0.004	0.009	0.015	0.58	10
	SAD	Varian TrueBEAM Stx	0.809	1.918	0.530	0.361	0.077	0.041	0.371	0.029	0.077	0.046	1.1	10
	SSD	Varian TrueBEAM	0.792	2.236	0.600	0.351	0.082	0.088	0.961	0.059	0.135	0.102	1.0	11
**10 MV FFF**														
	SAD	Elekta Versa HD	0.900	1.602	0.591	0.133	0.166	0.094	0.201	0.046	0.06	0.170	0.57	10
	SAD	Varian TrueBEAM	0.773	2.844	0.588	0.266	0.200	0.015	0.088	0.005	0.009	0.026	0.56	10
	SAD	Varian TrueBEAM Stx	0.832	2.054	0.517	0.224	0.142	0.031	0.312	0.027	0.014	0.037	1.1	10
	SSD	Varian TrueBEAM	0.830	2.169	0.563	0.240	0.116	0.083	0.801	0.046	0.029	0.109	0.96	11
