# Supplementary material for: A multi‐institutional evaluation of small field output factor determination following the recommendations of IAEA/AAPM TRS‐483
Source: Med Phys. 2022 Jul 8;49(8):5537–50. doi: 10.1002/mp.15797 (PMC9541513; doi:10.1002/mp.15797)
Supplement: Supplementary file 2 — Supplementary material [file MP-49-5537-s005.pdf]

Table S2 Variation of Sclin measured by differnt centers using different detector combinations

Label	Linac Type	Energy	Setup	Detector Combination	Nom. FS/ cm	mean($S_{clin)}$ / cm	standard deviation(S_{clin}) / cm
1	Elekta Versa HD	6 WFF	SAD	PTW 31016 - PTW 60017 - PTW 60019	1.0	1.03	0.01
1	Elekta Versa HD	6 WFF	SAD	PTW 31016 - PTW 60017 - PTW 60019	2.0	2.03	0.03
1	Elekta Versa HD	6 WFF	SAD	PTW 60017 - PTW 60019	0.5	0.58	0.01
1	Elekta Versa HD	6 FFF	SAD	PTW 31016 - PTW 60017 - PTW 60019	2.0	2.03	0.01
1	Elekta Versa HD	6 FFF	SAD	PTW 60017 - PTW 60019	0.5	0.57	0.01
1	Elekta Versa HD	10 FFF	SAD	PTW 60017 - PTW 60019	0.5	0.58	0.01
1	Elekta Versa HD	10 FFF	SAD	PTW 60017 - PTW 60019	1.0	1.05	0.01
1	Elekta Versa HD	10 FFF	SAD	PTW 60017 - PTW 60019	2.0	2.04	0.01
3	Elekta Precise	6 WFF	SAD	PTW 60008 - PTW 60017 - PTW 60019	1.0	1.05	0.02
3	Elekta Precise	6 WFF	SAD	PTW 60008 - PTW 60017 - PTW 60019	2.0	2.03	0.04
3	Elekta Precise	6 WFF	SAD	PTW 60017 - PTW 60019	0.5	0.59	0.04
5	Elekta Versa HD	10 WFF	SAD	PTW 60012 - PTW 60019	0.5	0.73	0.06
5	Elekta Versa HD	10 FFF	SAD	PTW 60012 - PTW 60019	0.5	0.76	0.02
6	Varian TrueBEAM	6 WFF	SSD	PTW 31010 - Sun Nuclear EDGE	2.0	2.01	0.01
6	Varian TrueBEAM	10 WFF	SAD	IBA EFD3G - IBA/Wellhoefer CC01 - PTW 31014	2.0	2.00	0.01
7	Varian TrueBEAM Stx	6 FFF	SAD	IBA SFD - PTW 60019	0.5	0.54	0.02
8	SIEMENS Primus	6 WFF	SAD	PTW 31018 - PTW 60012 - PTW 60019	0.5	0.60	0.01
8	SIEMENS Primus	6 WFF	SAD	PTW 31018 - PTW 60012 - PTW 60019	1.0	0.92	0.01
8	SIEMENS Primus	6 WFF	SAD	PTW 31018 - PTW 60012 - PTW 60019	2.0	1.86	0.01
10	Varian 21EX	6 WFF	SSD	PTW 31006 - PTW 60008	1.0	1.16	0.01
10	Varian 21EX	6 WFF	SSD	PTW 31006 - PTW 60008	2.0	2.19	0.05
12	Varian TrueBEAM Stx	6 WFF	SAD	PTW 60017 - Sun Nuclear EDGE	1.0	1.09	0.01
12	Varian TrueBEAM Stx	10 WFF	SAD	PTW 60017 - Sun Nuclear EDGE	1.0	1.10	0.02
12	Varian TrueBEAM Stx	10 WFF	SAD	PTW 60017 - Sun Nuclear EDGE	2.0	2.11	0.01
12	Varian TrueBEAM Stx	10 FFF	SAD	PTW 60017 - Sun Nuclear EDGE	2.0	2.09	0.01
