# Supplementary figures and images for: A multi‐institutional evaluation of small field output factor determination following the recommendations of IAEA/AAPM TRS‐483
Source: Med Phys. 2022 Jul 8;49(8):5537–50. doi: 10.1002/mp.15797 (PMC9541513; doi:10.1002/mp.15797)

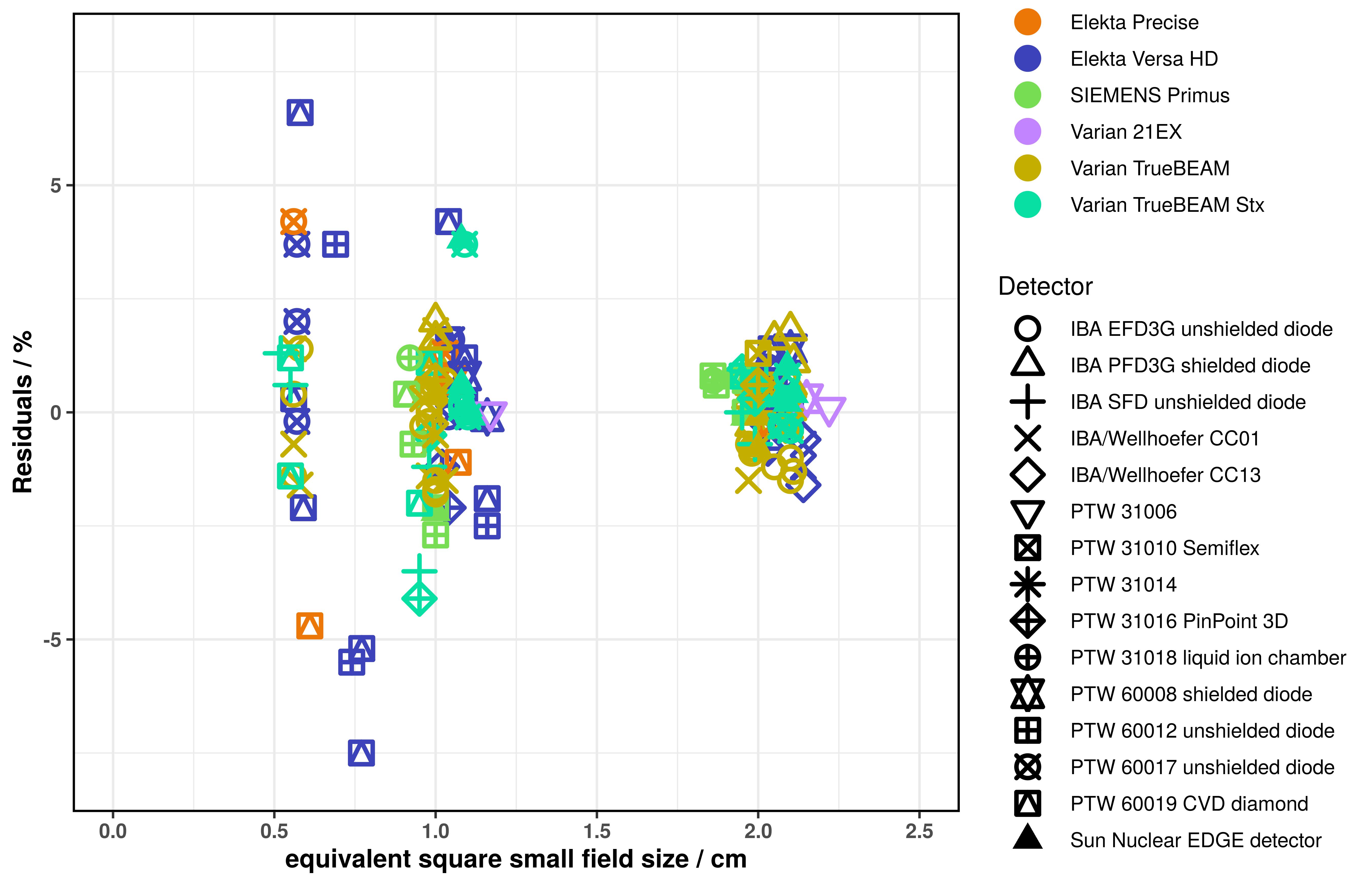

Supplement: Supplementary file 3 — Supplementary material [file MP-49-5537-s006.jpg]

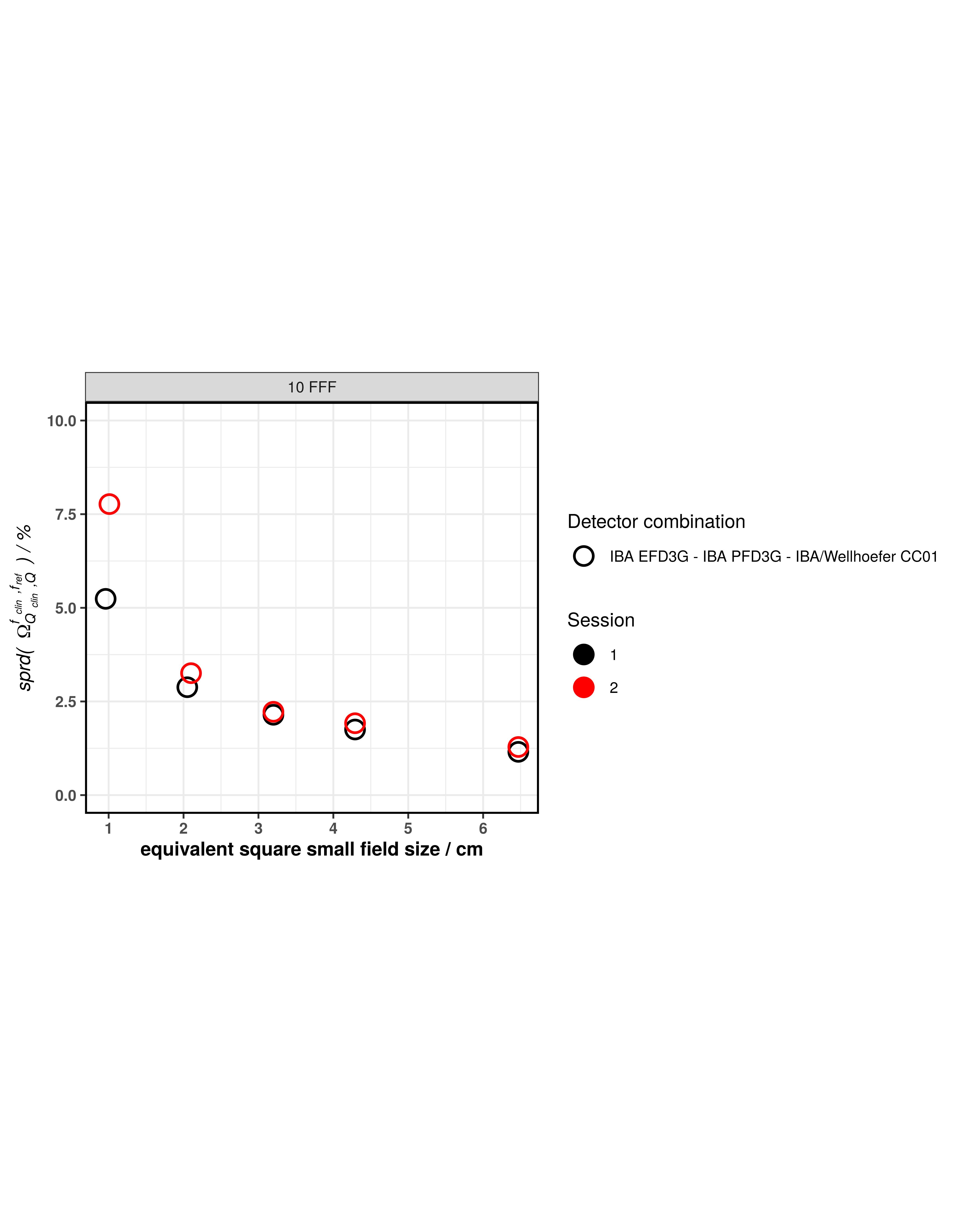

Supplement: Supplementary file 4 — Supplementary material [file MP-49-5537-s001.jpg]

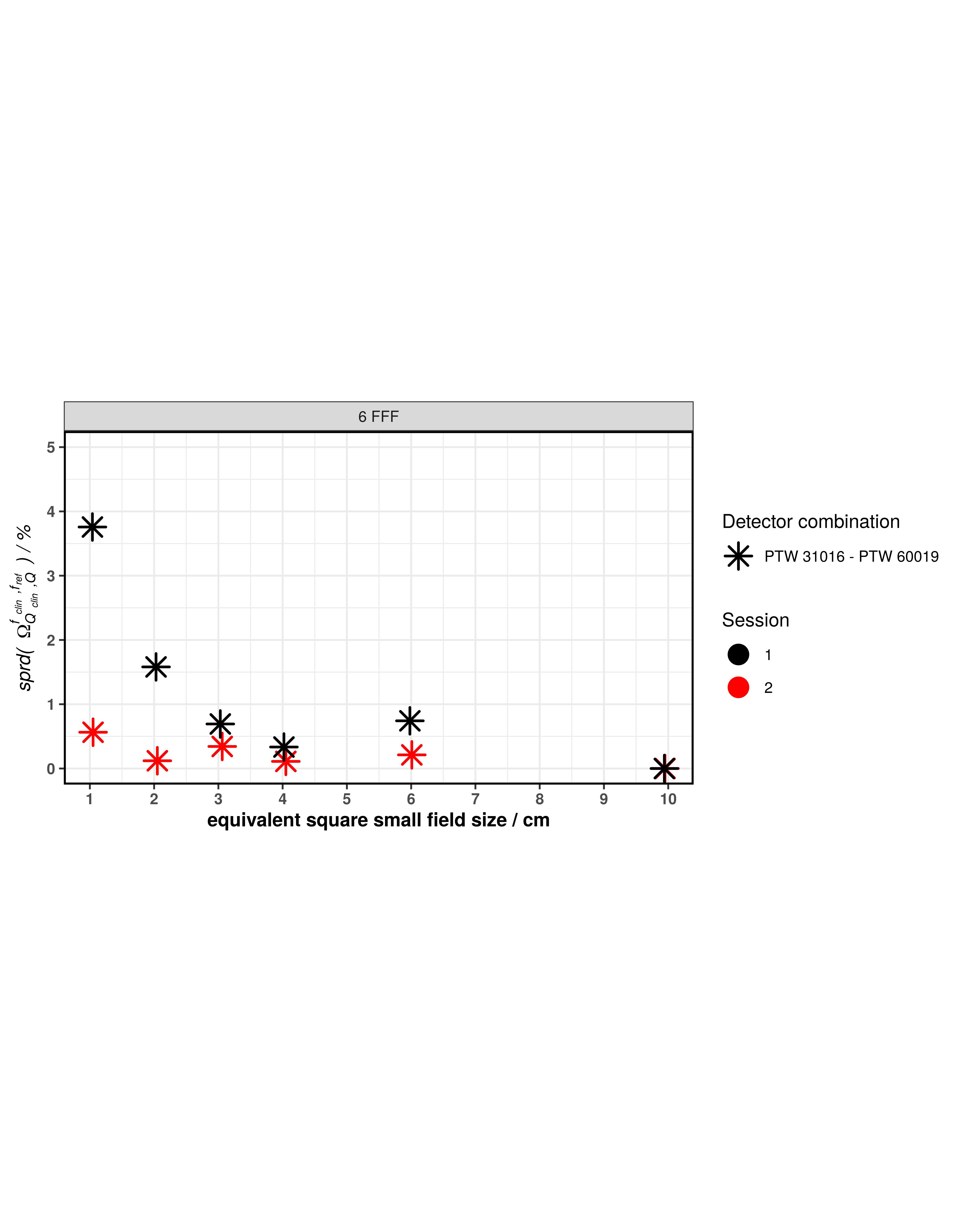

Supplement: Supplementary file 5 — Supplementary material [file MP-49-5537-s004.jpg]

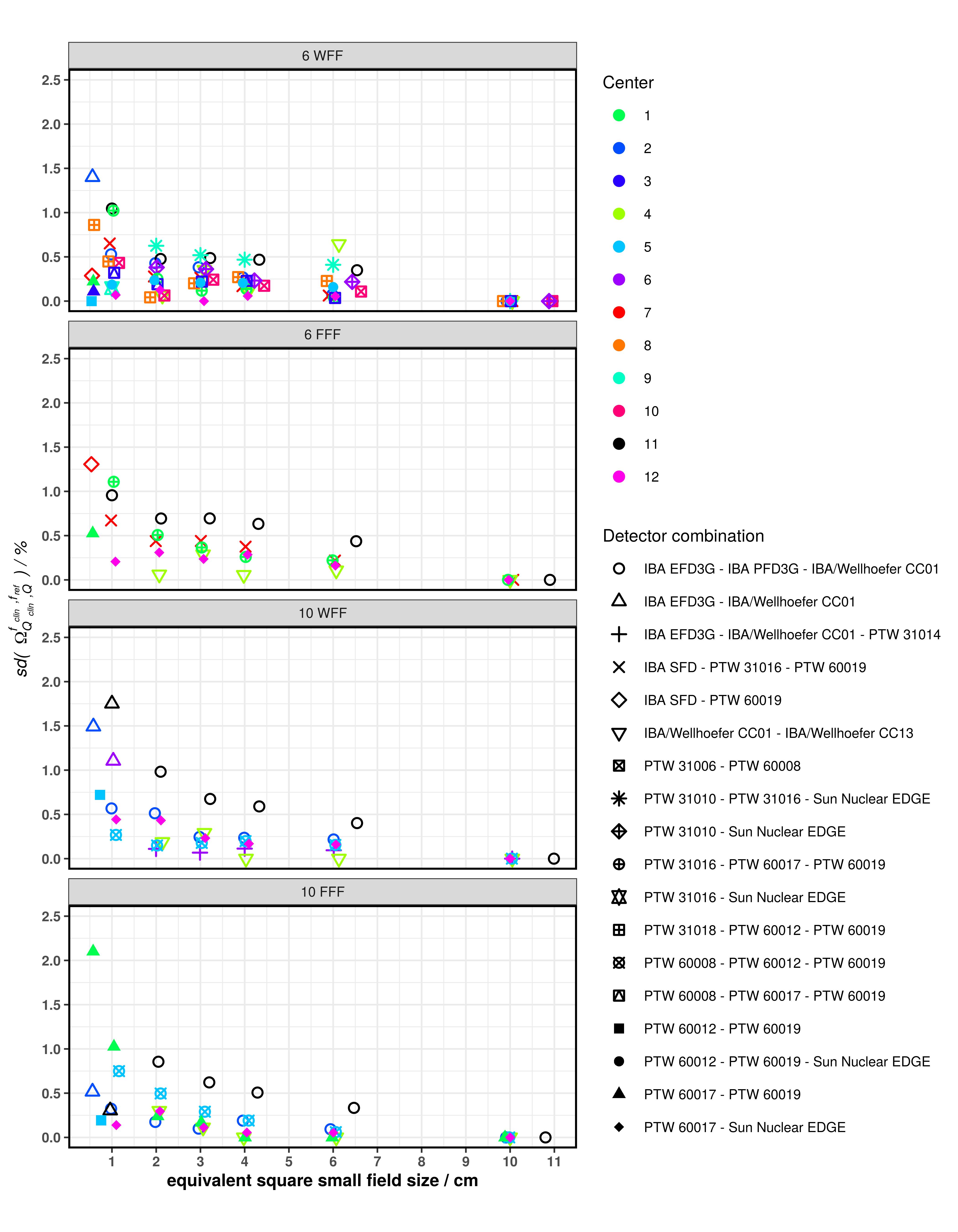

Supplement: Supplementary file 6 — Supplementary material [file MP-49-5537-s003.jpg]
